# PreImplantation Factor (PIF) promoting role in embryo implantation: increases endometrial Integrin-α2β3, amphiregulin and epiregulin while reducing betacellulin expression via MAPK in decidua

**DOI:** 10.1186/1477-7827-10-50

**Published:** 2012-07-12

**Authors:** Eytan R Barnea, David Kirk, Michael J Paidas

**Affiliations:** 1 SIEP - Society for the Investigation of Early Pregnancy, 1697 Lark Lane, Cherry Hill, NJ, 08003, USA; 2 BioIncept LLC, 1697 Lark Lane, Cherry Hill, NJ, 08003, USA; 3 Department of Obstetrics & Gynecology, University of Medicine and Dentistry of New Jersey- Robert Wood Johnson Medical School, Camden, NJ, USA; 4 PharmMedInfo, Poulsbo, WA, USA; 5 Yale Women and Children’s Center for Blood Disorders, Department of Obstetrics, Gynecology and Reproductive Sciences, Yale University School of Medicine, 333 Cedar St, PO Box 208063, New Haven, CT, 06520, USA

**Keywords:** Preimplantation Factor (PIF), Endometrium, Decidua, Gene Expression

## Abstract

**Background:**

Viable embryos secrete preimplantation factor (PIF), a peptide that has autocrine effects where levels correlate with cultured embryos development. sPIF (PIF synthetic analog) promotes implantation by regulating decidual-cells immunity, adhesion, apoptosis and enhances trophoblastic cell invasion. Herein sPIF priming effects on non-decidualized endometrium and decidualized-stroma are investigated, assessing elements critical for effective embryo-maternal cross-talk, prior to and at implantation.

**Methods:**

We tested sPIF effect on human non-pregnant endometrial epithelial and non-decidualized stroma α2β3 integrin expression (IHC and flow cytometry), comparing with scrambled PIF (PIFscr-control). We examined sPIF effect on decidualized non-pregnant human endometrial stromal cells (HESC) determining pro-inflammatory mediators expression and secretion (ELISA) and growth factors (GFs) expression (Affymetrix global gene array). We tested sPIF effect on HESC Phospho-kinases (BioPlex) and isolated kinases activity (FastKinase).

**Results:**

sPIF up-regulates α2β3 integrin expression in epithelial cells, (P < 0.05) while PIFscr had no effect. In contrast, in stromal cell cultures sPIF had no effect on the same. In HESC, sPIF up-regulates pro-inflammatory cytokines; IL8, IL1β and IL6 expression. The major increase in GRO-α, ICAM-1 and MCP-3 expression is coupled with same ligands secretion (P < 0.05). sPIF modulates in HESC GFs expression: up-regulates amphiregulin and epiregulin- critical for implantation and enhances several fibroblast growth factors (FGF) relevant for decidual function. In contrast, sPIF down-regulates major pro-proliferative ligands, betacellulin and IGF1 expression. sPIF modulatory effect on GFs is exerted by down-regulating pro-proliferative phospho-activated MAPkinases, p-MEK1 and p-ERK (P < 0.01, P < 0.04, respectively). Stress-induced p-38-MAPK (P = 0.04) and c-Jun kinase signaling involved MAPK8IP2 (−2.1 fold) expression decreased which protects against reactive oxygen species. Although pro-inflammatory p-NFkB (P = 0.06) decrease was mild, its promoter TNFRS11 expression markedly (−25-fold) decreased. In contrast, anti-proliferative phosphatases PTPRZ1 and PPP2R2C expression increased.

**Conclusions:**

sPIF post-fertilization primes endometrial-epithelium, while during implantation creates a beneficial pro-inflammatory milieu. PIF acts by balancing decidual pro-implantation properties while controlling excessive pro-proliferative and inflammatory signals expression. Overall, PIF influences critical peri-implantation events in a sequential coordinated fashion which facilitates embryo implantation.

## Background

Embryo-maternal cross talk is essential for establishment of normal pregnancy and such is critical at earliest stages - post-fertilization [1-4]. At that time, contact between embryo (segregated in the zona-pellucida) and maternal environment is limited. The pre-implantation period could be perceived as silent; when the maternal system is unaware of the embryo presence. However, recent data reported by us and by others indicates that already post-fertilization and prior to implantation, embryo-maternal dialogue critical for impending pregnancy develops [5-10]. Effect of numerous compounds that promote a receptive endometrium was previously examined. The role of cytokines/chemokines, integrins, and growth factors (GFs) was tested. Specifically EGF-related compounds have been shown to have contrasting effects. EGF for example promotes murine implantation while blocking decidualization in culture [11]. Further, trophoblast-conditioned media reduces TGFβ2 and IGF1 secretion in decidualized-cells [12].

In this context, we focused on preimplantation factor (PIF), an embryo-specific peptide secreted by viable mammalian embryos starting at two-cell stage and throughout viable pregnancy, absent in non-viable embryos [4-6,13-18]. PIF levels in culture, correlate with embryo viability [19,20]. PIF exhibits a direct autotrophic effect, promotes embryo development in culture and negates maternal serum-induced toxicity derived from patients with history of recurrent pregnancy loss [19,20]. PIF is expressed by the placenta and present in maternal circulation until term. PIF acts mainly on activated immunity creating a Th2/Th1 bias thus modulating maternal immune system without suppression [8,21]. Similarly, in non-pregnant autoimmune models, short-term, low-dose sPIF (synthetic PIF analog) prevents juvenile diabetes development by restoring pancreatic function [22] and in a harsh neuroinflammation model reverses inflammation while promoting neural repair [23]. sPIF promotes first trimester cultured trophoblast cells invasion, facilitating placental engraftment and development, while not synergizing with epidermal growth factor (EGF), a non pregnancy–specific proliferation promoting growth factor that is expressed in the endometrium [24]. sPIF also conditions the intrauterine environment, particularly the sex steroid-primed human endometrial stromal cells (HESC), and the first trimester decidua in culture by enhancing embryo implantation and providing support for the embryo by acting in the first trimester decidua. sPIF effect is exerted by modulating local immunity, adhesion and controlling apoptosis evidenced by affected genes expression and associated tissue proteome [25].

Since PIF is already secreted shortly post-fertilization, a 5–7 day delay exists until implantation takes place. We queried whether PIF can prime the endometrium during that critical period. We also examined PIF role in affecting pro-inflammatory factors secretion by HESC. sPIF promotes trophoblast invasion- essential for proper placental development independent of EGF. Therefore sPIF effect on endogenous (GFs) expression including EGF and regulation by phosphorylated MAP-kinases activity was examined. Such information establishes PIF’s critical role in creating a pro-receptive environment for the embryo prior and during implantation.

Herein, we examine sPIF effect on non-pregnant, non-decidualized endometrium, evaluating α2β3 integrin expression, a prime implantation marker [3]. Further, sPIF effect on HESC, examines integrins and pro-inflammatory genes expression and secretion. We also determine sPIF effect on relevant endogenous GFs expression in HESC and down-stream pathways involved in their regulation. Such data is relevant for understanding embryo-maternal communication at earliest stages of pregnancy.

## Methods

### Peptide synthesis

Synthesis was previously described [25]. In short, synthetic PIF analog (MVRIKPGSANKPSDD) or MVRIKPGSA and scrambled PIF (GRVDPSNKSMPKDIA) PIFscr – used as negative control, were produced by using solid-phase peptide synthesis (Peptide Synthesizer; Applied Biosystems, Foster City, CA) employing Fmoc (9-fluorenylmethoxycarbonyl) chemistry [21,24,26]. Final purification was carried out by reversed-phase high-pressure liquid chromatography, and peptide identity was verified by mass spectrometry [25].

### Endometrial cell cultures

Approval by the Institutional Review Board at the Yale University School of Medicine and St. Mary Hospital, Waterbury, CT, was obtained to collect endometrium and to carry out the endometrial experiments in this study. The method was previously published [25,27]. Briefly, discarded endometrial tissues from premenopausal women that undergo hysterectomies due to benign indications (primarily myomas) were analyzed. Women provided consent via the standard hospital consent form. There were no anticipated risks associated with participation in this research over the risks associated with the surgical procedure that women underwent for removal of their uterus. After removal during hysterectomy, the uterus was evaluated in the Department of Pathology at Yale New Haven Hospital and in St. Mary Hospital, CT, Dr. M. Albini. The pathologist provided a sample of discarded endometrial tissue, transported to our research laboratory for sPIF experiments. Collected endometrial tissues 400 mg were digested with 0.1% DNAase 0.005%/1h/37C with gentle mixing [28,29]. Following sedimentation of glandular structures and separation of ESC, the glands were purified of ESC and macrophage contaminants were removed by incubation at 37°C in a Falcon flask. This was followed by a second enzymatic digestion isolating the individual endometrial epithelial cells (EEC) from the glands for flow cytometric measurements. Glands were digested with trypsin (0.25%)–EDTA (0.03%)–DNase (0.1%) for 10 min at 37°C. Subsequently EEC were washed with DMEM–fetal bovine serum (FBS, 2%) and filtered out through a 37 μm mesh sieve. Stromal and epithelial cell dispersions were counted in a haemocytometer and cell viability was assessed using Trypan Blue exclusion method. ESC and EEC percent viability was >80%. Polyclonal antibodies to vimentin (Vm) and cytokeratin (Ck) were used to examine the homogeneity of ESC (Vimentin+) and EEC (Cytokeratin+) in cell smears of cellular dispersions. The maximum cross-contamination between ESC and EEC was <1%.[30]. Subsequently cells were cultured separately in cell bottom plates (8 well) for 5 days in DMEM-MCDB105 medium with 10% FCS (Sigma Chemical Co., St. Louis, MO) and for 24hrs in serum-free media containing 1–125 nM sPIF (15, or 9 aa). Epithelial and stromal were detached with Trypsin 0.01% EDTA 1nM (10min) and washed with PBS and cells were counted. Cells were stained with an anti-α2β3 integrin mAb (clone SSA6, 1:100) and an anti-mouse-FITC conjugate (1:100). α2β3 integrin expression was then determined by flow cytometry. A negative control with nonspecific IgG isotype-matched mAb (Dako, Carpinteria, CA) and an anti-HLA-I mAb (clone w6/32, 1:100) were introduced in each determination (n = 5). α2β3 integrin concentrations Mean ± SEM) were expressed as percentage of basal antigen expression in epithelial cells without sPIF. *P* < 0.05 was considered statistically significant. The sPIF-induced effect was compared to cells exposed to BSA 2%, 10% FCS, or PIFscr 1-500nM used as controls.

In other experiments endometrial cells were isolated and resuspended in RPMI-1640, grown to confluence, leucocyte free (<1%). After reaching confluence, cells were decidualized using 10^-8^ M estradiol and 10^-7^ M of the synthetic progestin analogue R5020 (Du Pont/New England Nuclear Products, Boston, MA). Cells were switched to defined medium containing insulin, transferrin, and selenium, (Collaborative Research, Inc., Waltham, MA) with 5 μM trace elements (GIBCO), and 50 μg/ml ascorbic acid (Sigma-Aldrich) and treated overnight with sPIF (100 nM) or vehicle only. The media and tissues were collected and frozen at −80°C. Products secreted into the media were analyzed using the bead-analysis kit (Bio-Rad, CA) and the lysates phospho-proteins by BioPlex kit (Bio-Rad, CA) in triplicate determining (Mean±SEM) activity, following the manufacturer instructions. sPIF and PIFscr effect (10 nM- 10mg/ml) on isolated kinases was tested by using the proprietary Fastkinase method analyzing direct action of peptides generating IC50 data, XlFit by software (MDSPS, Bothell, WA).

### Microarray analysis

Total RNA was extracted from each cell culture as previously reported [25]. Analysis of HESC ± sPIF 100 nM (n = 3/group) was carried out by using Affymetrix (Santa Clara, CA) U133 Plus 2.0 Array (18,400 human genes), followed by fluorescence labeling and hybridization with Fluidics Station 450 and optical scanning with GeneChip Scanner 3000 (Affymetrix) at W. M. Keck Foundation Biotechnology Resource Laboratory, Yale University, New Haven, CT. Raw data were analyzed by GeneSpring software (Agilent, Santa Clara, CA), normalized for interchip and intrachip variation to eliminate false-positive results. Microchip was analysed by National Institutes of Health’s ImageJ image-analysis software (http://rsb.info.nih.gov/ij). P < 0.05 and two fold change was considered statistically significant. See Additional file 1: Table S1 describes signal intensity and P value associated with gene detection.

### Statistical analysis

Data was analyzed using Meta-plex software, t-test; significance was set at p < 0.05. The Bioplex data was analyzed by using log transformed data, followed by ANOVA P < 0.05. For gene experiments the data were first analyzed using Student’s t-test with P < 0.05 results followed by fold change testing with a cutoff equal or >2-fold reported.

## Results

### sPIF up-regulates cultured endometrial epithelial cells α2β3 integrin

**Figure 1:**
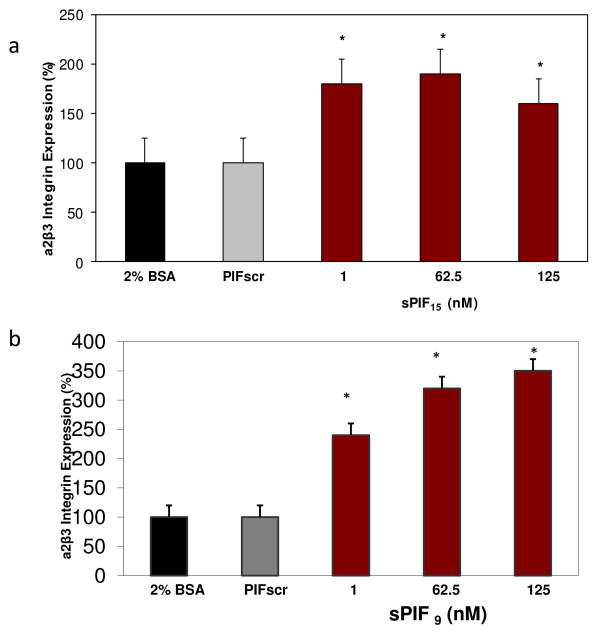
**sPIF promotes endometrial receptivity by up-regulating α2β3 integrin.** Endometrial epithelial cells were isolated and cultured for 5 days in presence of FCS followed by 24hrs in serum-free media containing 1–125 nM sPIF (15, or 9 aa). At the end of culture cells stained with specific antibody and α2β3 integrin expression was then determined by flow cytometry. Nonspecific IgG isotype served as negative control. **a**) PIF_15_ promotes α2β3 integrin in a dose dependent manner as compared with scrambled PIF 125nM or 2%BSA, effect was not significant. **b**) PIF_9_ promotes endometrial-α2β3 integrin in a dose dependent manner as compared with 2%BSA, control. Data expressed as (Mean ± SEM.) percent change in basal antigen expression in epithelial cells as compared to negative controls. Data are representative of three different experiments with similar results (ANOVA, *P < 0.05).

sPIF effect on endometrial receptivity was investigated modeling the early luteal phase at a stage when circulating estrogen and progesterone levels are low (no direct embryo-endometrial contact). For this end, primary epithelial and stromal endometrial cells cultured for five days reaching confluence were used, exposing them to sPIF for 24hrs. Two different versions of sPIF were tested, both found in the embryo culture media, having the same core 9 first aminoacids. It was found that in epithelial cell cultures both, sPIF(15aa) and sPIF (9aa) at low physiologic concentrations increased α2β3 integrin, an important marker of endometrial receptivity, up to 4-fold in a dose-dependent manner (P < 0.01). (Figure 1a,b) Two different complimentary modalities of analysis were conducted; first qualitative analysis using an anti-α2β3 integrin-antibody carrying out staining of epithelial cells at 5 days of culture. Second, this was corroborated by a semi-quantitative analysis using flow cytometry (FC). The data showed that sPIF is most effective at low concentrations similar to physiologic range found in pregnancy. Significant stimulatory effect was noted with both ligands already at a low 1nM concentration, effect being dose dependent. sPIF specificity was confirmed since PIFscr (scrambled peptide, same amino acids as sPIF but in different order), bovine serum albumin (2% BSA), or 10% FCS effect tested in parallel and used as controls, had no effect. Importantly, the increase seen in α2β3 integrin in epithelial cells was not replicated by exposure to primary culture of non-decidualized stromal cells when tested with two independent methods (not shown). Overall, these experiments indicated that sPIF promotes a critical pro-implantation marker in uterine epithelium independently of sex-steroid exposure.

### sPIF effect on integrins in HESC

The endometrial epithelial cells are the site where embryo-derived signaling primes post-fertilization to promote implantation. sPIF increased α2β3 integrin in epithelial cells in primary culture and not in non-decidualized stromal cells. Therefore, we inquired whether sPIF also has similar effect on integrins in HESC, which are estrogen and progestin-induced decidualized stromal cells. sPIF in HESC as expected, does not affect ITGB3 expression the gene that encodes for α2β3 integrin, however, sPIF promotes ITGA2- (1.96 fold), a platelet membrane integrin. An increase in ITGB1(1.92, fold) a fibronectin receptor which is activated by Netrin-4 involved in vascular development was also noted. In contrast, sPIF down-regulated ITGA9 (−2.1 fold), expression, a receptor for VCAM1, cytotactin and osteopontin which binds to VEGF and promotes adhesion and cell migration. Thus, we showed that sPIF effect is dynamic and modulates the endometrium in a different manner, dependent on the evolving stage of embryo-maternal cross-talk at pre-and during implantation period.

### sPIF promotes pro-inflammatory mediators secretion by HESC

**Figure 2:**
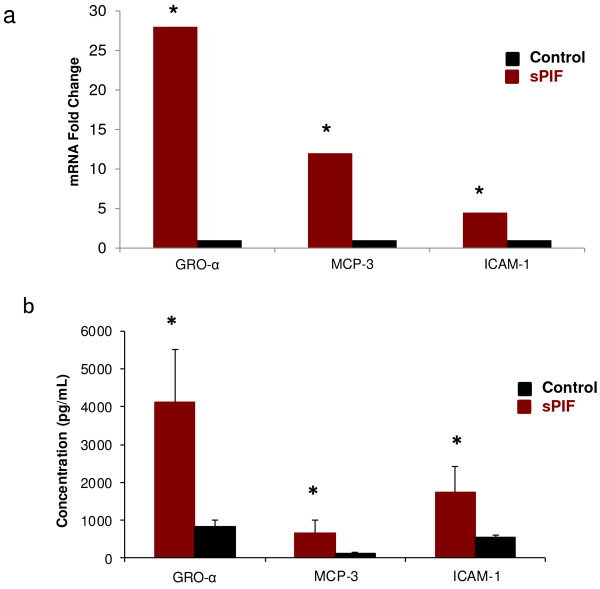
**sPIF-induced promotion of pro-inflammatory genes is coupled with their secretion.** HESC were cultured in presence with 100 nM sPIF for 24 hours. Triplicate cells were extracted and global mRNA was.analyzed (Affymetrix). Gene expression >3 fold was considered significant. Culture media of HESC was collected and pro-inflammatory ligands was analyzed using ELISA. **a**) sPIFpromotes mRNA expression of pro-inflammatory cytokines and adhesion molecules as compared to controls. (**b**) PIF promotes inflammatory cytokine and chemokine secretion by HESC, P < 0.05. The increase in gene expression was confirmed by increased secretion into the media.

In line with the described beneficial pro-inflammatory effects of sPIF on HESC genes expression, we examined whether they are also translated into changes in secreted pro-inflammatory mediators- relevant for uterine environment conditioning. There was a significant up-regulation of major inflammatory interleukins, IL8 (82-fold) IL1β (7-fold) and IL6 (10.7-fold). The increase in intercellular adhesion molecule 1, (ICAM-1, CD54) gene expression was coupled with ligand secretion, a leukocyte adhesion protein (integrin alpha-L/beta-2). The chemokines (growth-related oncogene-α (GRO-α, CXCL1) gene expression chemotactic for neutrophils, was coupled with protein secretion. The monocyte chemotactic protein-1 (MCP-1, CCL7) gene expression was also coupled with protein secretion. (Figure 2a,b) We show that sPIF-induced gene expression leads to pro-inflammatory ligands secretion favorably conditioning the uterine environment for embryo implantation.

### sPIF up-regulates amphiregulin, epiregulin and FGFs expression in HESC

We reported that sPIF did not synergize with exogenously added EGF in promoting trophoblast invasion. Since EGF is a member of a major proliferation-promoter family expressed by HESC, we examined sPIF effect on EGF and related GFs expression. Amphiregulin and epiregulin genes expression increased which encode proteins that promote decidual and cultured embryos development. In contrast, pro-proliferative betacellulin expression decreased an EGF receptor ligand as well as IGF1 while TGFβ2 decreased mildly. (Table 1) HBEGF (heparin binding) and EGF gene itself or its receptor expression was not affected. Significantly, increased expression of several fibroblast growth factors (FGF) was observed (Table 2). sPIF exerts a highly selective modulatory effect on HESC, balancing decidual pro-implantation properties while controlling excessive pro-proliferative signals expression.

**Table 1:** PIF modulates growth factors to promote endometrial receptivity

**Up-Regulated**		**Down-Regulated**	
Amphiregulin	8.1	Betacellulin	9.5
Epiregulin	11.1	IGF1	2.2
		TGFβ2	1.6

HESC Fold Change in Gene Expression.

**Table 2:** PIF promotes fibroblast growth factor expression

**FGF**	**Fold Change**
2	2
5	4
11	4.1
13	11.5
14	2.2

In HESC – Global Gene Analysis.

### sPIF modulates GFs by down-regulating p-MAPK and up-regulating phosphatases expression

**Figure 3:**
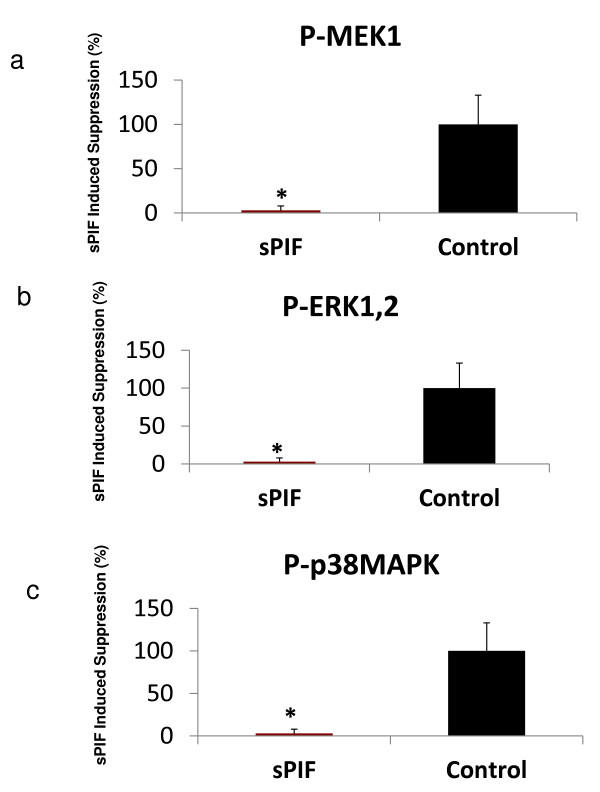
**sPIF down-regulates activated p-MAPK involved in HESC proliferative pathway.** HESC were cultured in presence of 100nM sPIF for 24 hours at the end of culture lysates phospho-proteins were analyzed by using BioPlex kit in triplicate determining (Mean+/−SEM), P < 0.05. **a**)p-MEK-1 **b**) p-ERK1,2, **c**) p-p38MAPK. The decrease in all three kinases was marked as compared to PBS control.

Since sPIF modulates GFs in HESC, we aimed to determine whether it is exerted by regulation of downstream transcription factors. Kinases added a phosphate molecule (serine, thrionine, or tyrosine) are activated. sPIF markedly down-regulates critical transcription factors involved in proliferative pathways (Figure 3a,b,c). A number of activated-kinases activity involved in MAPK pathway decreased; two proliferation promoters; ERK- activator kinase (p-Thr202, Tyr204) -ERK1/2, (P < 0.01) which was followed downstream by a major decrease in p-MEK1 (p-Ser 218, Ser 222), (P < 0.04). In addition to the decrease in TGFβ2 expression, p-38-MAPK (p-Thr180, Tyr182), (P < 0.04) also decreased, involved in response to stress stimuli. There was also a significant decrease in MAPK8IP2 (−2.1 fold) (MAPK8 interacting protein-2) expression which encodes a scaffold protein that mediates c-Jun amino-terminal kinase signaling pathway, protecting against reactive oxygen species. The mild decrease in pro-inflammatory p-NFkB (P = 0.06) was accentuated with a major decrease in TNFRS11 expression, (−25-fold) and FAS (−3.3 fold) major NFkB activators, while p-IKB, and p-AKT were not affected by sPIF. Upon examining associated phosphatases gene expression that inactivate kinases, it was found that the phosphatases PTPRZ1 (phosphacan, 5.5-fold) which interacts with P53-a major proliferation controller, and PPP2R2C (2.9-fold) increased, both of which inhibit MAPK and proliferative pathways. Thus sPIF has a dual coordinated effect on both kinases and phosphatases. In addition, these pathways are directly involved in down-regulation of both proliferation and inflammation promoting pathways in HESC.

### sPIF effect on MAPK does not involves their catalytic site

**Figure 4:**
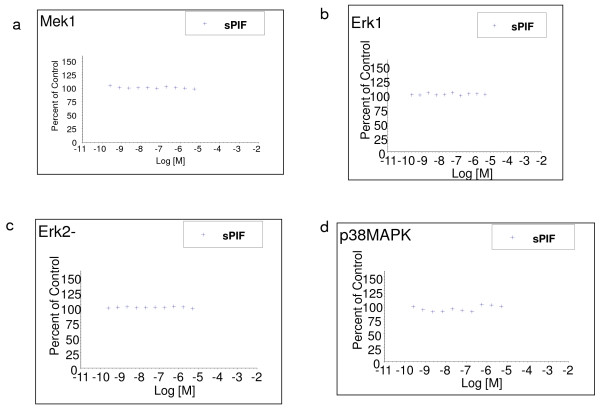
**sPIF does not directly affect MAPK activity.** sPIF at different concentrations was added to purified enzymes in presence of enzyme substrate determining IC50 values. In all tested enzymes, sPIF did not interfere with the enzyme activity. **a**) MEK-1 **b**) ERK-1, **c**) ERK-2. **d**) p38-MAPK.

We found that sPIF modulates differentially endogenous GFs expression and this is associated with regulation of MAPK pathways. To examine whether sPIF has a direct effect on MAP kinases enzymes catalytic activity, the Fastkinase method was used to evaluate a given ligand effect on the enzyme activity itself as part of a panel. In contrast to the marked inhibitory effect on phosphoproteins expression seen in HESC, sPIF did not affect recombinant kinases involved in the MAPK pathway ERK1,2, MEK-1, p-38-MAPK, or NFkB activity *in vitro* (Figure 4). sPIF also did not affect directly the EGF protein tyrosine kinase activity, the down-stream activator of the growth factor. Thus, sPIF down-stream inhibitory effects on proliferative elements in HESC is exerted by down-regulation of pro-proteins involved in the MAPK pathway, which is independent of a direct action on the catalytic site of these enzymes.

## Discussion

PIF is a specific embryo-derived biomarker which, through autocrine effects, supports embryo development and survival starting prior implantation. In addition, sPIF promotes embryo receptivity creating a favorable uterine environment acting on decidualized stromal cells (HESC) and first trimester decidua [26].

We herein demonstrate that sPIF has an evolving coordinated pro-receptive effect on uterine environment favoring embryo implantation and development. This is evidenced by the noted increase in α2β3 integrin - critical pro-implantation marker in non-pregnant endometrial epithelial cells. In HESC- an implantation model sPIF modulates integrins expression and increases pro-inflammatory ligands expression and secretion- creating a pro-receptive uterine milieu. Effect of sPIF on HESC supports implantation by increasing amphiregulin, epiregulin and FGFs while reducing proliferation promoter, betacellulin expression. Mechanistically, sPIF modulating effect on GFs involves p-MAPK inhibition coupled with increased phosphatases expression.

To demonstrate our observations we used a multifaceted approach. Specifically, we: 1) Tested effect of sPIF on primary endometrial and stromal cell cultures α2β3 integrin. 2) Analyzed sPIF effect in HESC on integrin expression. 3) Correlated effect of sPIF on HESC pro-inflammatory genes expression with secreted ligands. 4) Examined sPIF effect on endogenous GFs expression in HESC. 5) Determined sPIF effect on GFs-related phospho-kinases and associated phosphatases in HESC.

Prior to this study, it was not fully established how early embryo-maternal cross talk is initiated. We herein report for the first time, that PIF, an embryo-specific compound, has an important evolving role in uterine priming prior embryo arrival to the uterus. The sPIF-induced up-regulation of α2β3 integrin, a critical implantation marker in primary epithelial cells correlates with window of implantation [3]. Consequently, sPIF effect already initiates post-fertilization when the embryo is segregated within the fallopian tube and no direct contact between embryo and uterus exists. The sPIF-induced increase in α2β3 integrin is independent of estrogen and progesterone exposure since steroids were not added in culture when PIF itself is added. Such is compatible with the post-fertilization period when circulating progesterone levels are low. Therefore PIF-induced endometrial priming precedes that induced by progesterone and it evidences that an embryo-specific signal is needed for effective endometrial-priming. Both embryo-secreted PIF peptides15aa and shorter (9aa) are present in embryo culture media [21,26] had similar promoting effect which supports their pro-receptive properties. The 15aa is the dominant peptide secreted by the embryo therefore it was used in subsequent experiments. Lack of effect of scrambled PIF and negative controls tested in parallel confirmed sPIF specificity. Notably, sPIF did not affect primary stromal cells (non-decidualized) evidencing that increased α2β3 integrin expression is induced only in epithelial layer, first contact between embryo and endometrium.

The HESC model was chosen since it mimics the implantation decidua. Here, stromal cells conditioned by sex steroids replicate mid-luteal phase when circulating progesterone and estrogen peak and implantation takes place. Precise mechanisms involved in decidualization have not been previously fully characterized [31]. In HESC sPIF affects integrins involved in inflammation and vascular pathway [32] acting on Netrin-4, an integrin receptor [33] while the gene specific for α2β3 integrin expression is not affected. Increase in integrin A2 may protect against fetal loss due to platelet membrane polymorphism [34]. The shift between pre-and implantation with respect to integrin expression reflects PIF evolving role at this critical period.

sPIF modulates HESC genes and several associated proteins. Whether such leads to pro-inflammatory ligands secretion creating a favorable uterine milieu was examined. sPIF markedly increases several critical pro-inflammatory genes expression which in the innate arm of immunity, is involved in monocytes recruitment and in adaptive immunity in leukocyte recruitment and adhesion [9]. Endometrial-biopsy performed in IVF patients also increased local macrophage presence, GRO-α and other inflammatory mediators and led to improved pregnancy outcome [35]. We now document that such an increase in pro-inflammatory ligands can also be induced in HESC by PIF an embryo-specific signal.

Sex steroids, especially estrogen, condition endometrial stromal cells towards decidualization by exerting potent-proliferative effects. sPIF promotes cultured trophoblastic cells invasion in culture, not amplified by EGF, a potent non-pregnancy specific growth factor. The major decrease noted in the expression of betacellulin, a promoter EGF, supports the anti-proliferative role of PIF. Endogenous GFs expression is significant in HESC, therefore it is remarkable that sPIF increases amphiregulin and epiregulin, factors that support embryo maturation and decidual development [36]. This is of importance since amphiregulin is not a progesterone-dependent uterine gene [37]. sPIF promoted decidual fibroblast growth factors (FGFs) expression - some of them are novel and such is relevant since the stroma is composed of fibroblasts [31]. FGFs affect embryo implantation and support improved endometrial-trophoblastic interaction [38,39]. Thus sPIF shifts the decidua from a proliferative to a receptive mode needed for implantation.

GFs proliferative effects are exerted through activated phospho-kinases. The consistent and profound down-stream inhibition induced by sPIF on HESC MAPK pro-proliferative pathways support a targeted effect on GFs expression. The decrease in p-MEK1 a kinase up-stream of p-ERK1 might have led to the down-stream inhibition as well as may lead to B-Raf kinase inhibition [40]. Reduction in p-38-MAPK inhibits TGFβ2-induced signaling and the decrease in c-Jun expression reflects anti-inflammatory effects of sPIF. Inhibition of the NF-kB pathway directly interferes with the pro-inflammatory TNF-induced signaling [41]. Based on our results sPIF inhibitory action on MAPK is independent of their catalytic site. sPIF increased kinase-inactivating phosphatases the first enzyme removes Ser/Thr phosphate [42] while PTPRZ1, a tyrosine phosphatase, has differentiating effect in the nervous system, and was not previously reported in the decidua [43,44]. Through coordinated effect on p-MAPK and phosphatases PIF shifts the endometrial stroma from a proliferative pro-inflammatory into a receptive environment critical for embryo survival.

It is recognized that in order to implant, the embryo induces specific changes in the endometrium absent in non-pregnant decidua [45,46]. Trophoblast-conditioned media affects a large number of decidual genes where specific factor(s) involved were not identified [12]. Role of various non-embryo specific factors involved in decidual function was also recently reviewed [2]. We focused herein on PIF as a single embryo-specific viability signal and its effect on the endometrium, demonstrating wide-range regulatory effects.

The strength of this study is use of sPIF in two complementary models, examining post-fertilization endometrial environment and sex steroid-conditioned stromal cells- implantation model. Also, testing critical integrins, pro-inflammatory mediators secretion, endogenous GFs expression and mechanistically examination of activated kinases and phosphatases involved in their regulation. The study is limited since observations in culture do not necessarily reflect the *in vivo* condition.

## Conclusions

Our findings support the premise that modulation of maternal uterine environment is significantly embryo-driven. Modulation starts shortly post-fertilization and PIF plays a critical evolving role amplified during implantation. By priming the uterus PIF creates a receptive milieu for embryo development which may have significant therapeutic implications.

## Competing interests

PIF is a patented compound owned by BioIncept, LLC., Dr. Eytan R. Barnea, is its (uncompensated) Chief Scientist. Dr. J.H. Barnea, is its (uncompensated) President and CEO, and majority shareholder. Yale University (Dr. Michael Paidas) received an unrestricted grant from BioIncept. All authors, including Dr. David Kirk, declare no conflict of interest.

## Authors’ contributions

MJP carried out the endometrial cultures. DK participated in fast kinase analysis. ERB analyzed and wrote the paper. All authors read and approved the final manuscript.

## Supplementary Material

Additional file 1 **Table S1.** Gene expression data, intensity and statistics. Click here for file
