# Supplementary material for: PreImplantation Factor (PIF) promoting role in embryo implantation: increases endometrial Integrin-α2β3, amphiregulin and epiregulin while reducing betacellulin expression via MAPK in decidua
Source: Reprod Biol Endocrinol. 2012 Jul 12;10:50. doi: 10.1186/1477-7827-10-50 (PMC3444419; doi:10.1186/1477-7827-10-50)
Supplement: Additional file 1 — Table S1. Gene expression data, intensity and statistics. [file 1477-7827-10-50-S1.docx]

| Supplemental Table SI: Gene expression data, intensity and statistics | | | | |  |
| --- | --- | --- | --- | --- | --- |
| **Gene** | **Intensity** | **P value** | **Intensity** | **P value** |  |
| TFGB2 | 44 | 0.6 | 5 | 0.4 |  |
| Betacellulin | 36 | 0.03 | 15 | 0.69 |  |
| IGF1 | 1138 | 0.002 | 564 | 0.0024 |  |
| Amphiregulin | 50 | 0.001 | 406 | 0.002 |  |
| Epiregulin | 10 | 0.3 | 111 | 0.007 |  |
| FGF13 | 3 | 0.6 | 39 | 0.001 |  |
| FGF2 | 1362 | 0.0002 | 2704 | 0.0002 |  |
| FGF5 | 22.2 | 0.39 | 92 | 0.046 |  |
| FGF11 | 61 | 0.27 | 252 | 0.046 |  |
| FGF14 | 46 | 0.01 | 103 | 0.001 |  |
| ITGA9 | 67 | 0.03 | 32 | 0.056 |  |
| MAPK8 | 124 | 0.01 | 60 | 0.08 |  |
| TNFRSF11A | 29 | 0.03 | 1.4 | 0.19 |  |
| PTPRZ1 | 5 | 0.46 | 28 | 0.01 |  |
| PPP2R2C | 12.4 | 0.14 | 37 | 0.002 |  |
| IL8 | 312 | 0.0002 | 21133 | 0.0002 |  |
| IL1b | 104 | 0.2 | 720 | 0.0008 |  |
| IL6 | 195 | 0.0002 | 2095 | 0.0002 |  |
| ICAM1 | 123 | 0.14 | 589 | 0.002 |  |
| MCP 3 | 35 | 0.03 | 439 | 0.002 |  |
| Groa | 99 | 0.008 | 2806 | 0.0002 |  |

Following global HESC gene analysis each gene was examined for intensity of expression (absence/presence) and the associated P value was determined by setting significance at <0.05. (Fold change described in Tables I, II, and result section. Housekeeping genes served as internal controls B2M, GADPH, RPL35A, HNKPD, RPL9.
